# Chronic lymphocytic leukemia at ASH 2017

**DOI:** 10.1007/s12254-018-0414-0

**Published:** 2018-06-01

**Authors:** David Wanner, Michael Steurer

**Affiliations:** Medical University Innsbruck, Innsbruck, Austria

**Keywords:** Chronic lymphocytic leukemia, Ibrutinib, Venetoclax, ASH

## Abstract

At ASH (American Society of Hematology) 2017 three out of a plethora of trials showed remarkable and promising results. The combinations of venetoclax with rituximab and ibrutinib with venetoclax convinced with striking efficacy together with a manageable safety profile in relapsed/refractory setting as well as in first line therapy of high-risk disease. These two combinations are potential new standard treatment options in chronic lymphocytic leukemia.

## Introduction

Over the last years the development of novel agents like ibrutinib, idelalisib and venetoclax led to a paradigm shift in the treatment of chronic lymphocytic leukemia (CLL). Immunochemotherapy regimens are now more and more being replaced by targeted therapies due to superior efficacy and better safety profiles, in particular for patients with relapsed disease [1–4]. Of the plethora of clinical data presented at the last annual meeting of the American Society of Hematology (ASH) this article will focus on two relevant trials: the MURANO trial setting a new standard of care for relapsed/refractory disease and the CLARITY trial pointing out future developments for the treatment of CLL.

## Venetoclax plus rituximab is a new standard of care for relapsed/refractory CLL

The MURANO trial, presented by John Seymour et al., compared the efficacy and safety of the Bcl2-inhibitor venetoclax in combination with rituximab to the combination of bendamustine and rituximab in patients with relapsed/refractory CLL. In this multicenter phase III trial *n* = 389 patients were randomized to receive either venetoclax plus rituximab or bendamustine plus rituximab. Inclusion criteria comprised prior treatment with 1–3 lines of therapy (including ≥1 chemo-containing regimen), age over ≥18 years and a response duration of at least 24 months if prior treatment included bendamustine. Primary endpoint was the local investigator (INV) assessed progression-free survival (PFS), while secondary endpoints include the review committee (IRC) assessed complete response rate (CR), the overall response rate (ORR) and overall survival (OS) as well as IRC assessed PFS and MRD(minimal residual disease)-negativity. The safety profile was also a key endpoint of the trial. In the venetoclax plus rituximab arm, venetoclax was administered at a dosage of 400 mg daily after standard dose escalation (“ramp-up”) over 5 weeks. Rituximab was given over 6 cycles with an initial dosage of 375 mg/m^2^ at cycle 1, followed by 500 mg/m^2^ at cycles 2–6. In the control arm bendamustine and rituximab (BR) were administered at standard dosage of 70 mg/m^2^ and 375 mg/m^2^, respectively (cycles 1–6).

The INV and IRC ORR were both significantly higher in the VenR (venetoclax+rituximab) arm (93.3% vs. 67.7%, *p* ≤ 0.0001 and 92.3% vs. 72.3%, *p* ≤ 0.0001). Median PFS was 18.1 months in the BR arm and has not been reached in the VenR arm (*p* < 0.0001). Subgroup analyses revealed no significant differences in PFS even in the presence of high-risk markers such as del (17p), TP53 mutations or unmutated IGHV status.

With the exception of neutropenia (57.7% vs. 38.8%) the experimental arm was superior concerning the occurrence of grade 3–4 AEs. Tumor lysis syndrome (TLS), a well-known complication of the initial phase of venetoclax treatment, could be effectively prevented by mandatory safety measurements as given in the protocol including hydration, use of allopurinol ± rasburicase, and meticulous laboratory controls. TLS occurred in 3.1% (vs. 1.1% with BR) and had clinical significance in one patient in each treatment arm (acute renal failure and transient increase of creatinine values, respectively) [5].

Summing up, treatment with VenR represents a new standard of care for relapsed/refractory CLL. Efficacy is significantly superior to immunochemotherapy with BR and it displays a favorable safety profile. Based on these results regulatory submissions to health authorities are underway and a wider indication for the use of venetoclax in CLL can soon be expected.

## Ibrutinib plus venetoclax for relapsed/refractory CLL

Another novel treatment strategy presented at ASH is the combination of the Btk-inhibitor ibrutinib with venetoclax. Hillmen et al. (CLARITY trial) as well as Jain et al. explored the therapeutic potential and safety profile of this combination in relapsed/refractory CLL and first line in high-risk CLL patients, respectively. The rationale behind this therapeutic approach is based on the different modes of action of these two drugs and the fact that ibrutinib enhances the sensitivity of CLL cells to venetoclax [6]. This sensitization is at least in part mediated by the ibrutinib-induced increased expression of BIM (Bcl-2-like protein) in CLL cells. BIM is a member of Bcl-2 protein family and it has been shown to interact with other members of the Bcl-2 group, including Bcl-2 and MCL-1, while acting as an apoptotic promoter [7]. As mentioned above, the varying efficacy of these drugs in different CLL compartments, i. e., peripheral blood, bone marrow and lymph nodes, is another rationale for this new combination. Ibrutinib unfolds its impact very effectively primarily in lymph nodes and the bone marrow leading to a lymphocyte mobilization from the protective microenvironment of the lymphatic tissues rendering CLL cells more susceptible to the action of venetoclax. Venetoclax, in turn, is highly effective in the peripheral blood [8], thereby, enhancing the effects of ibrutinib in a synergistic fashion.

The preliminary presentation of the phase I/II CLARITY trial comprised *n* = 54 patients with refractory/relapsed CLL. Baseline characteristics included a median age of 64 years (range 31–84 years), 74% suffered from unmutated CLL and 20% were positive for a del (17p). The participating patients had received a median of one prior line of therapy (range 1–6). In all, 81% had been pretreated with FCR (fludarabin, cyclophosphamide, rituximab) or BR, whereas idelalisib had been administered in 20%.

Patients are treated with ibrutinib at a standard dosage of 420 mg per day for 8 weeks followed by the addition of venetoclax, applying the usual weekly dose escalation up to 400 mg daily. MRD is assessed every 3 months in peripheral blood (PB) and after 6, 12 and 24 months in bone marrow (BM). If MRD negativity is achieved (including PB and BM) after 6 or 12 months, venetoclax will be stopped, whereas ibrutinib is to be continued. If no sustained MRD negativity is achieved venetoclax will be re-administered for another 24 months, and ibrutinib is given as long as clinically indicated.

The preliminary data presented at ASH 2017 showed the imposed results after treatment after 4 and 8 months, respectively (*n* = 38 patients reached at least the 8‑month cut off). ORR at 8 months was 100%, whereof 39% achieved CR, 8% CR with incomplete bone marrow recovery and the remaining 53% attained PR (partial remission). Concerning adverse events and toxicity neutropenia was as expected the most prevalent AE [9].

The second trial exploring the combination of ibrutinib and venetoclax is carried out by Jain et al. They presented early data on *n* = 116 patients of a phase I/II trial, including patients with relapsed/refractory CLL as well as untreated high-risk disease. High-risk disease was defined as at least one out of the following characteristics: age ≥ 65, del (11q), del (17p), mutated TP53 or unmutated IGVH status. In contrast to the CLARITY trial, patients receive ibrutinib for 28 days (4 weeks) before venetoclax dose escalation (max. 400 mg) is initiated. Ibrutinib is administered until disease progression and venetoclax will be given up to a maximum of 2 years. Primary endpoints are the CR as well as the CRi rates as defined by the IWCLL 2008 criteria. The baseline characteristics and the mutational status are mapped at Table 1.

**Table 1:** Baseline characteristics, IGHV, cytogenetic and molecular genetic mutational status [10]

Characteristics	Cohort 1	Cohort 2
*Median age, years (range)*	59 (32–76)	64.5 (35–82)
*Male, n (%)*	30 (81)	30 (75)
*Median prior tx, n (range)*	1 (1–4)	–
*FISH, n (%)*		
del (17p)	11 (30)	7 (18)
del (11q)	14 (38)	10 (25)
Trisomy 12	5 (14)	5 (12)
Negative	2 (5)	5 (12)
del (13q)	5 (14)	13 (33)
*Unmutated IGHV, n/N (%)*	27/31 (87)	30/37 (81)
*Cytogenetics n/N (%)*		
Complex	5/29 (17)	6/39 (15)
Diploid	10/29 (34)	16/39 (41)
*Mutations n/N (%)*		
TP53	10/32 (31)	7/40 (18)
NOTCH1	3/32 (9)	14/40 (35)
SF3B1	7/32 (22)	11/40 (28)
*ZAP-70 (>20% or IHC+)*	21/27 (78)	33/40 (83)
*CD38* *>* *30%*	22/36 (61)	23/40 (58)

*FISH* fluorescence in situ hybridization, *IGHV* immunoglobuline heavy chain variable region, *tx* therapy

At ASH the data of 77 patients, which were divided into two cohorts (cohort 1: relapsed/refractory CLL, cohort 2: 1st line treatment of high risk disease), were presented. Both cohorts showed impressive initial response rates (100%) as well CR rates of 77% (relapsed/refractory) and 80% (1st line), respectively. MRD negativity assessed by 4‑color flow with a sensitivity of 10^−4^ revealed increasing rates over time with a rate of 46% MRD negativity in cohort 1 and 100% in cohort 2 after treatment over 12 months. However, the impressive and promising results have to be interpreted with caution due to the low patient numbers (*n* = 5, *n* = 3 after 12 months). As in the CLARITY trial, neutropenia was the most common grade 3/4 adverse event (44 of 77 patients). Atrial fibrillation, a typical AE caused by ibrutinib, was the second most AE occurring in 10 out of 77 patients. Concerning TLS only 2 cases of laboratory TLS were observed [10].

Summing up, the combination of ibrutinib and venetoclax represents an attractive novel treatment option in CLL for both relapsed/refractory and previously untreated disease. In particular the high rates of MRD-negativity achievable even in high-risk patients is promising potentially allowing for deeper responses, longer response duration and treatment-free intervals, respectively. However, this concept has to be further explored in larger, randomized trials.

In conclusion, the clinical data presented at ASH 2017 show that CLL treatment is moving away from immunochemotherapy regimens towards targeted treatment with novel agents. Whereas drugs like ibrutinib and idelalisib have already improved the outcome of many CLL patients in particular of those with high-risk disease, novel combinations are being developed as shown in the trials discussed above. Moreover, interesting new drugs (e. g., CC-122, second generation Btk/PI3K inhibitors) and cellular therapies (CAR T‑cells) are also coming up making the future of CLL patients look even brighter.
